# The characteristics of cerebrospinal fluid tumor microenvironment in a patient with leptomeningeal metastases from cancer of unknown primary

**DOI:** 10.1016/j.gendis.2023.04.026

**Published:** 2023-06-23

**Authors:** Haoyu Ruan, Zhe Wang, Xuemei Tang, Qiong Zhan, Kun Chen, Lu Gao, Ming Guan

**Affiliations:** a Department of Laboratory Medicine, Huashan Hospital, Fudan University, Shanghai 200040, China; b Department of Laboratory Medicine, The First Affiliated Hospital of Nanjing Medical University, Nanjing 210029, China; c Department of Physiology, Naval Medical University, Shanghai 200433, China; d Department of Oncology, Huashan Hospital, Fudan University, Shanghai 200040, China

Cancer of unknown primary (CUP) is a rare disease characterized by metastases in which the primary tumor is of unknown origin. The cerebrospinal fluid (CSF) tumor microenvironment of CUP is still unknown. A Chinese male was diagnosed with leptomeningeal metastases from CUP (CUP-LM) based on the following medical examination results: partial leptomeningeal enhancement by brain magnetic resonance imaging, few malignant cells of diverse morphology in CSF, and no abnormalities or lymphadenopathy by systemic examination. The CSF tumor microenvironment was analyzed by single-cell RNA sequencing (10× genomics). A total of 3346 cells of high quality were enrolled for analysis and classified into eight major cell types. The CSF tumor microenvironment of the CUP-LM case showed CD8^+^ T cells in a dysfunctional state, an increased proportion of regulatory T cells (Treg) and LAMP3-positive dendritic cells, which helped shape an immunosuppressive landscape. In addition, intensive communications between CD4_Treg and other cell subtypes were identified from aspects of inhibitory, costimulatory, or chemokine communications. The tumor cells enhanced the immunosuppressive tumor microenvironment by the interaction of co-inhibitory checkpoints with the tumor-infiltrating immune cells.

A 47-year-old Chinese male was referred to our hospital for headache, dizziness, and projectile vomiting. Brain magnetic resonance imaging showed partial leptomeningeal enhancement and suspicious abnormal enhancement in the left temporal lobe; thus, we considered the possibility of metastases. CSF evaluation showed moderate pleocytosis with nucleated cells of 40 × 10^6^/L, clear and transparent, total protein of 0.81 g/L (normal range: 0.15–0.45 g/L), lactate of 3.2 mmol/L (1.0–2.8 mmol/L), glucose of 3.28 mmol/L (2.5–4.4 mmol/L), and chloride ion of 122 mmol/L (120–130 mmol/L). CSF morphological assessment discovered a few malignant cells of diverse morphology (Fig. S1A). Immunohistochemical studies failed to define the tumor origin. Laboratory investigations of the CSF showed that alpha-fetoprotein, carcinoembryonic antigen, carbohydrate antigen 125 (CA12-5), CA15-3, CA19-9, CA72-4, cytokeratin-19-fragment antigen 21-1, and neuron-specific enolase levels were within normal limits. Squamous cell carcinoma antigen (normal range: 0.0–2.7 ng/mL) levels were up-regulated in the CSF (20.63 ng/mL) and serum (4.29 ng/mL). We performed a systemic evaluation in order to detect the primary lesion. Physical examination showed no abnormalities and no lymphadenopathy. FDG-PET/CT detected fluorodeoxyglucose uptake in the ascending colon, which was most likely due to polyps and inflammation. A colonoscopy showed inflammation of the rectal mucosa and multiple rectal polyps which were diagnosed as (rectal) adenomatous polyps by pathological examination. Capsule endoscopy showed inflammation of the small intestinal mucosa and no obvious space-occupying lesions. Gastroscopy suggested chronic superficial gastritis with erosions. Systemic evaluation could not identify the primary tumor lesion; hence the patient was diagnosed with CUP-LM.

We generated scRNA-seq (10× genomics) profiles of CSF cells from the CUP-LM patient. Eight major cell types (3346 cells) were identified on a uniform manifold approximation and projection (UMAP) plot, including T cell (T, 2230 cells), natural killer cell (NK, 74 cells), B cell (B, 188 cells), dendritic cell (DC, 452 cells), monocyte (Mono, 205 cells), macrophage (Mac, 154 cells), and tumor cell (Tumor, 43 cells) clusters (Fig. 1A, B).

**Figure 1:**
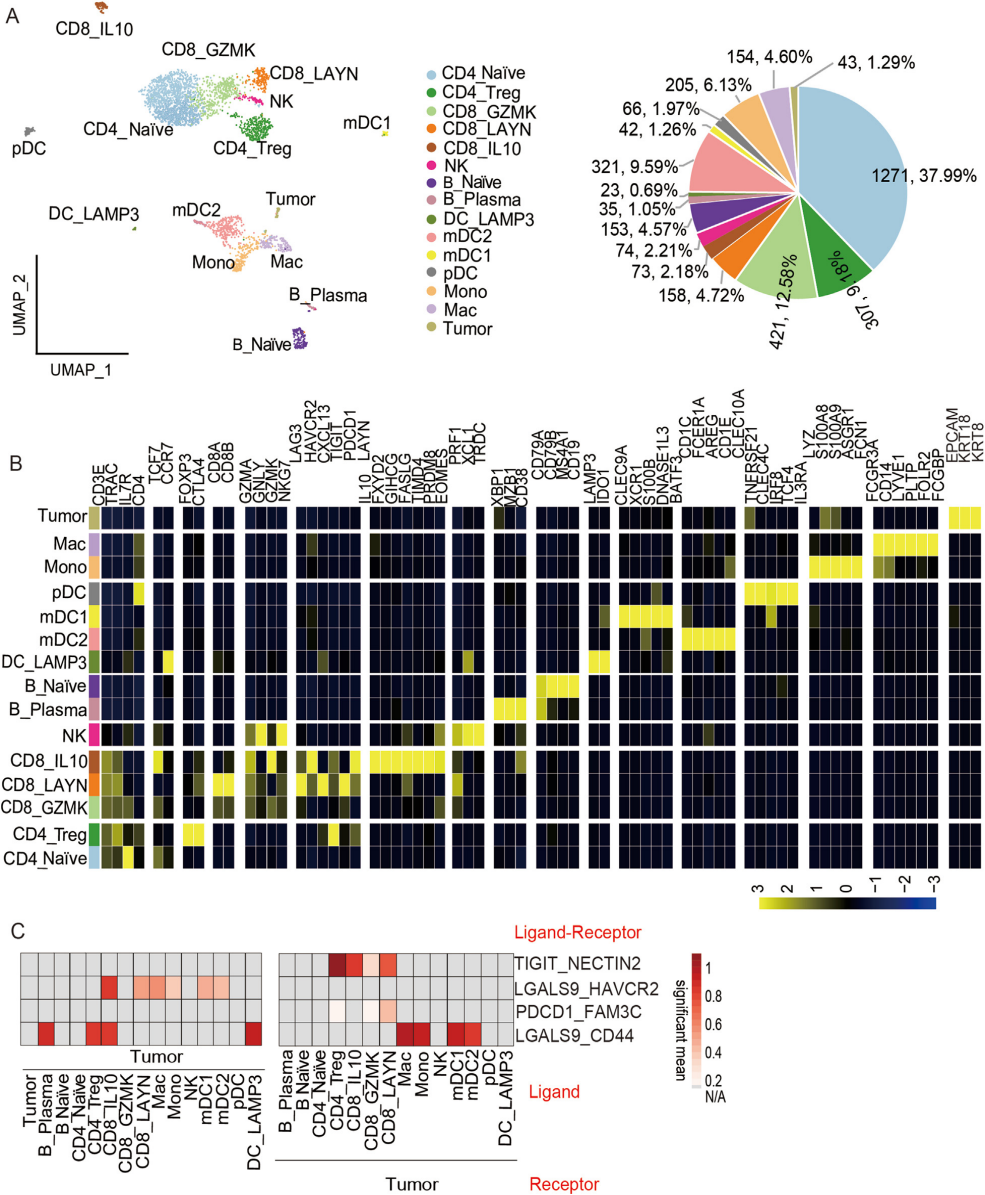
single cell RNA sequencing profiling of the CSF tumor environments of the CUP-LM patient. **(A)** The UMAP plot, showing celltypes (Left) and the proportion of celltypes (Right) in CSF of the CUP-LM patient. **(B)** Markers labeling celltypes in the UMAP plot. Celltypes: CD4_ Naïve, Naïve CD4 T cells; CD4_Treg, regulatory CD4 T cells; CD8_GZMK, GZMK positive CD8 T cells; CD8_LAYN, LAYN positive CD8 T cells; CD8_IL10, IL10 positive CD8 T cells; NK, Natural Killer cells; B_ Naïve, Naïve B cells; B_Plasma, Plasma cells; DC_LAMP3, LAMP3 positive Dendritic Cells; mDC2, myeloid DC type 2; mDC1, myeloid DC type 1; pDC, Plasma DC; Mono, monocytes; Mac, Macrophages; Tumor, tumor cells. Canonical markers: T-Cell, CD3E, TRAC, IL7R, CD4-T, CD4; CD4_Naïve: TCF7, CCR7; CD4_Treg: FOXP3, CTLA4; CD8_GZMK: GZMA, GNLY, GZMK, NKG7; CD8_LAYN: LAG3, HAVCR2, CXCL13, TIGIT, PDCD1, LAYN; CD8_CD10: IL10, FXYD2, GIHCG, FASLG, TIMD4, PRDM8, EOMES; NK: PRF1, XCL1, TRDC; B_plasma: XBP1, MZB1, CD38; B_naïve: CD79A, CD79B, MS4A1, CD19; DC_LAMP3: LAMP3, IDO1; mDC2: CD1C, FCER1A, AREG, CD1E, CLEC10A; mDC1: CLEC9A, XCR1, S100B, DNASE1L3, BATF3; pDC: TNFRSF21, CLEC4C, IRF8, TCF4, IL3RA; Mono: LYZ, S100A8, S100A9, ASGR1, FCN1; Mac: FCGR3A CD14, PLTP, LYVE1,FOLR2, FCGBP; Tumor (epithelial): EPCAM, KRT18, KRT8. T exhaustion signature: PDCD1, HAVCR2, LAG3, TIGIT, CTLA4, LAYN, EOMES; T cytotoxicity signature: GZMA, GNLY, GZMK, IFNG, NKG7, naïve signature: CCR7, TCF7. **(C)** Heatmap showed selected ligand–receptor interactions between tumor cells and other celltypes. CSF, cerebrospinal fluid; CUP-LM, cancer of unknown primary of leptomeningeal metastases.

The T-NK cells were classified into three subtypes of CD8^+^ T cell (CD8_GZMK, CD8_LYAN, and CD8_IL10), two CD4^+^ T cell subtypes (CD4_ Naïve, CD4_Treg), and one NK cell subtype (Fig. 1A, B). CD8_GZMK cluster (421 cells) presented a lower cytotoxicity signature and exhaustion signature than CD8_LYAN (158 cells) cluster and CD8_IL10 cluster (Fig. 1B; Fig. S1B). CD8_IL10 cluster (73 cells) showed higher expression of IL10, FXYD2, GIHCG, TIMD4, PRDM8, lower cytotoxicity compared to other T-cell clusters (Fig. 1B; Fig. S1B). The cytotoxic markers were co-expressed with inhibitory molecules, suggesting a gradual rather than binary state of T cell dysfunction in the CUP-LM patient. In addition, compared to normal CSF sample,^1^ CD8^+^ T cells in the CUP-LM CSF showed a higher exhaustion signature (but no significant difference in cytotoxicity signature (Fig. S1C)) and a higher proportion of Tregs (307/2230, 13.76% *vs*. 1.89% ± 0.62% in controls; data were represented as mean ± standard deviation), indicating an immunosuppressive environment (Fig. 1A).^1^ NK cluster (74 cells, 2.21%) was classified as a cytotoxic NK subtype (Fig. 1A, B; Fig. S1B). B cells (188 cells, 5.62%) could be divided into plasma B cells (B_ plasma) and naïve B cells (B_ naïve) (Fig. 1A, B). Compared to normal CSF samples,^1^ naïve B cells were elevated in the CUP-LM patient (4.57% *vs*. 0.39% ± 0.24% in controls; data were represented as mean ± standard deviation), and the emergence of plasma cells (1.05%) suggested an activated B cell response.

The average proportion of myeloid cells was 24.24%, and four distinct subsets of dendritic cells (DC) were identified, namely, DC_ LAMP3 (23 cells), two myeloid DC clusters (mDC1, 42 cells, and mDC2, 321 cells), and one plasmacytoid DC cluster (pDC, 66 cells; Fig. 1A, B). The emergence of a rare population, DC_ LAMP3, which expressed a higher level of immune regulatory genes (IL4I1, SOCS1, and CD200), indicated the inhibition of CD8^+^ T cells directly or via recruitment of Tregs^2^ (Table S1).

Intensive communications across CSF cell clusters were characterized by the CellPhoneDB package (Fig. S1D). We focused on the main cellular communications between CD4_Treg and other components in terms of inhibitory, costimulatory, or chemokine communications (Fig. S2). For chemokine communications, the DC_LAMP3 subset expressed CCL22 and CCL17, which can bind to CCR4 to recruit Tregs into the tumor microenvironment.^3^ CCL19-CCR7 and CCL19-CXCR3 axes were also active in the interaction between DC_LAMP3 and CD4_Treg. CD4_Treg contacted with CD8_IL10 and NK dependent on the CCR3-CCL28 loop, indicating potential chemotaxis between CTL/NK cells and Tregs. For costimulatory communications, LAMP3_DC had the potential to contact Tregs via the TNFSF4–TNFRSF4 axis, which plays important roles in anti-tumor immunity of nasopharyngeal carcinomas.^3^ For inhibitory signals, PDCD1–CD274, PDCD1–PDCD1LG2, PDCD1–FAM3C, and TIGIT–NECTIN2 loops from Tregs to other immune cells were discovered. Treg-expressed CTLA-4 interacted with CD80/CD86 on antigen-presenting cells (Mac, mDC2, DC_LAMP3), and the costimulatory signal is a key mechanism of Treg-mediated suppression.^4^

We further characterized 43 tumor cells having epithelial marker expression. Sixty-three genes defining tumor clusters were selected (Table S2) and SCGB2A2 expression was positive in eight CSF tumor cells (Fig. S1F). In the CUP-LM case of our previous study, we discovered seven circulating tumor cells in CSF showing SCGB2A2 expression and discussed the diagnostic value of SCGB2A2 for tumor origin of breast cancer and salivary gland cancer.^5^ It is worth mentioning that we subsequently detected SCGB2A2 expression in CSF tumor cells of breast cancer (Fig. S1F).^1^ SCGB2A2 provided clues for the tumor origin of the CUP-LM patient, however, the patient was lost to follow up and a definitive conclusion failed to make. In addition, we focused on the communication between tumor cells and tumor-infiltrating immune cells (Fig. 1C). We identified the prominent co-inhibitory signal via the TIGIT–NECTIN2 and PDCD1_FAM3C axes between CD8^+^ T cells and tumor cells.

In conclusion, single-cell data of CSF cells may become a helpful molecular diagnostic way to help elucidate the primary tumor source of CUP in the future. Identification of the characteristics of CSF cells at the single-cell level could provide hints to illuminate the enigmatic biology of CUP-LM as well as potential targets for therapy.

## Ethics declaration

The proposed studies were approved by the Institutional Review Board of Huashan Hospital (HIRB, KY2019-002).

## Conflict of interests

The authors state no conflict of interests.

## Funding

This work was funded by the 10.13039/501100001809National Natural Science Foundation of China (No. 82102489, 82072367, 82120108011).

## Data availability

The original data of single-cell RNA sequencing of cerebrospinal fluid cells of the patient with leptomeningeal metastases from cancer of unknown primary are available in Table S4.
