# Supplementary material for: The characteristics of cerebrospinal fluid tumor microenvironment in a patient with leptomeningeal metastases from cancer of unknown primary
Source: Genes Dis. 2023 Jun 23;11(3):100992. doi: 10.1016/j.gendis.2023.04.026 (PMC10808954; doi:10.1016/j.gendis.2023.04.026)

Figure S1: The roles of the CSF cells in the immune functions of the CUP-LM patient.

(A) CSF morphological assessment showing few malignant cells of diverse morphology. Hematoxylin-eosin staining, magnification 1000×, scale bar represents 10 μm.

(B-C) Violin plot indicating the cytotoxic or exhausted score of sub-clusters of CD8+ T (CD8_GZMK, CD8_LAYN, CD8_IL10) and NK cells of the CUP-LM patient (B), CD8+ T cells from control samples (Control) and the CUP-LM patient (C). Wilcoxon Rank-Sum test, ***, p<0.001. Single-cell data of control CSF samples were obtained from GSE138266.

(D) Heatmap showing number of potential ligand-receptor pairs between clusters (Fig. 1A) predicted by CellphoneDB.

(F) The relative expression of SCGB2A2 in CSF tumor cells of breast cancer LM patients and the CUP-LM patient. Single-cell data of CSF samples from breast cancer LM patients were obtained from GSE150681 (BC-LM-B, BC-LM-C, BC-LM- E) and GSE202501 (BC-LM-F).

CSF, cerebrospinal fluid, CUP-LM, cancer of unknown primary of leptomeningeal metastases.


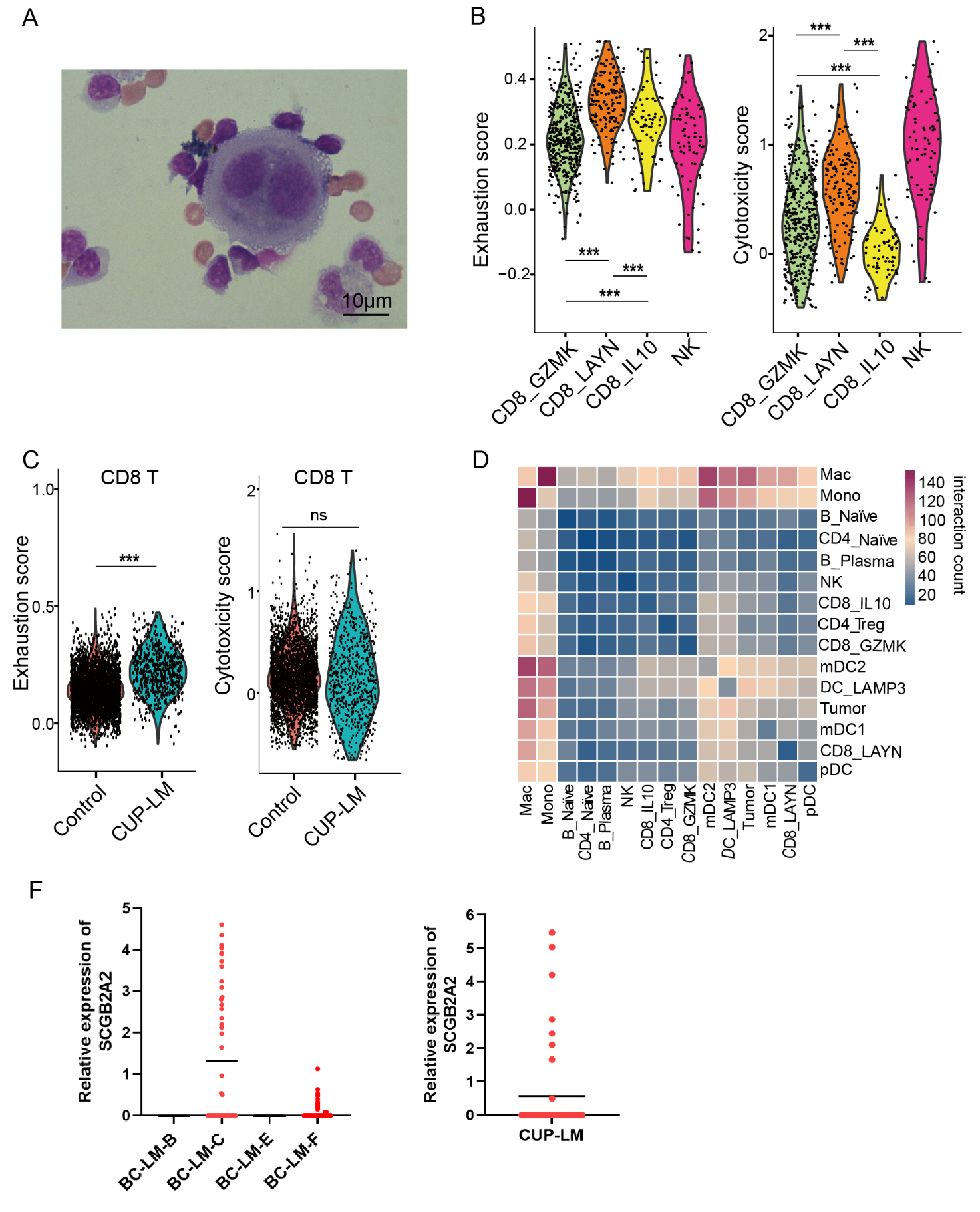

Supplement: Multimedia component 5 [file mmc5.docx]
