# Supplementary material for: The characteristics of cerebrospinal fluid tumor microenvironment in a patient with leptomeningeal metastases from cancer of unknown primary
Source: Genes Dis. 2023 Jun 23;11(3):100992. doi: 10.1016/j.gendis.2023.04.026 (PMC10808954; doi:10.1016/j.gendis.2023.04.026)

Figure S2. Heatmap showed selected ligand-receptor interactions between Treg and other components in terms of inhibitory, costimulatory, or chemokine communications. Celltypes: CD4_ Naïve, Naïve CD4 T cells; CD4_Treg, regulatory CD4 T cells; CD8_GZMK, GZMK positive CD8 T cells; CD8_LAYN, LAYN positive CD8 T cells; CD8_IL10, IL10 positive CD8 T cells; NK, Natural Killer cells; B_ Naïve, Naïve B cells; B_Plasma, Plasma cells; DC_LAMP3, LAMP3 positive Dendritic Cells; mDC2, myeloid DC type 2; mDC1, myeloid DC type 1; pDC, Plasma DC; Mono, monocytes; Mac, Macrophages; Tumor, tumor cells.


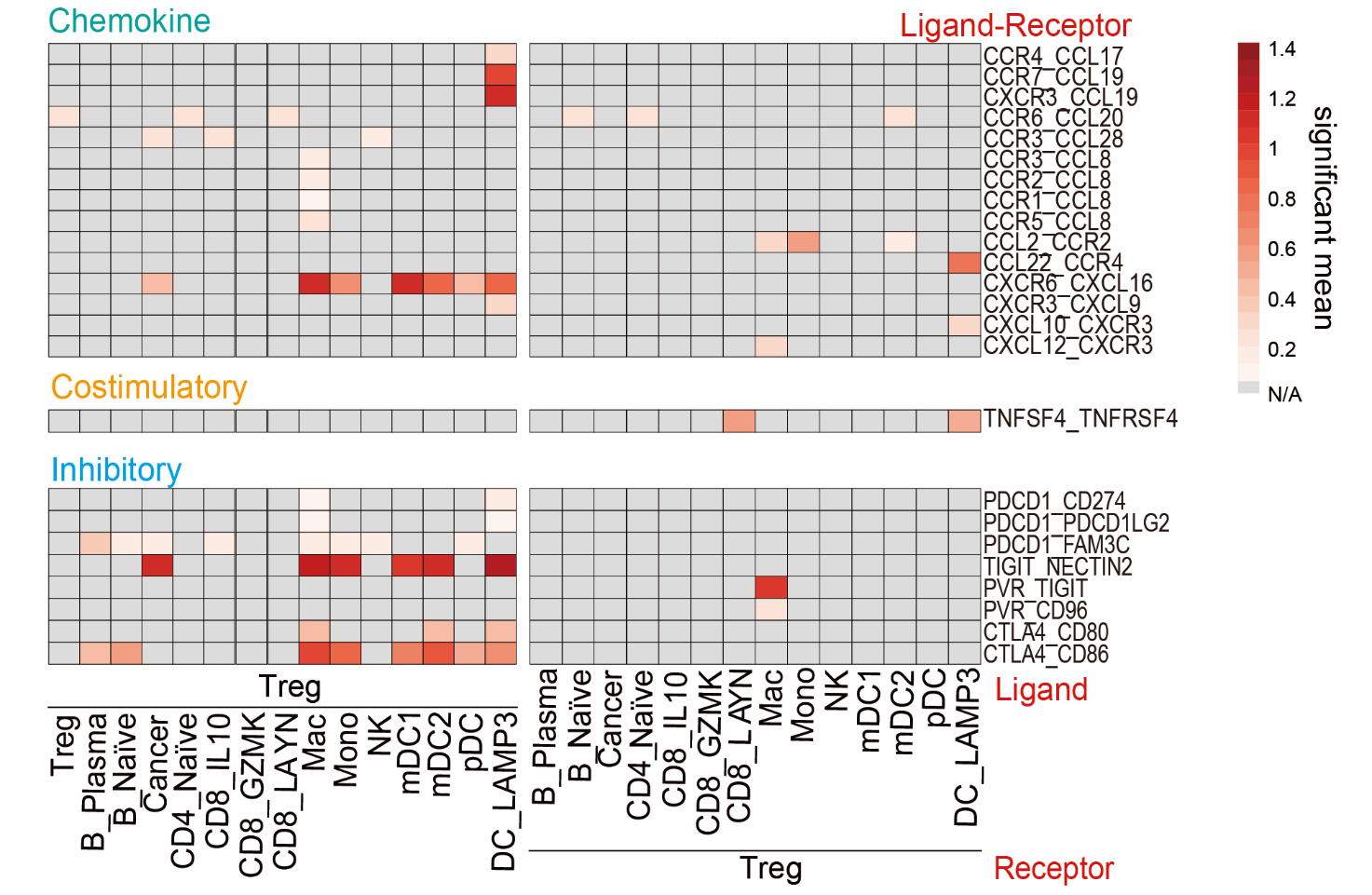

Supplement: Multimedia component 6 [file mmc6.docx]
